# Supplementary material for: Tracking karyotype dynamics by flow cytometry reveals de novo chromosome duplications in laboratory cultures of Macrostomum lignano
Source: Biol Open. 2026 Jan 23;15(1):bio062346. doi: 10.1242/bio.062346 (PMC12869490; doi:10.1242/bio.062346)
Supplement: Supplementary information [file biolopen-15-062346-s1.pdf]

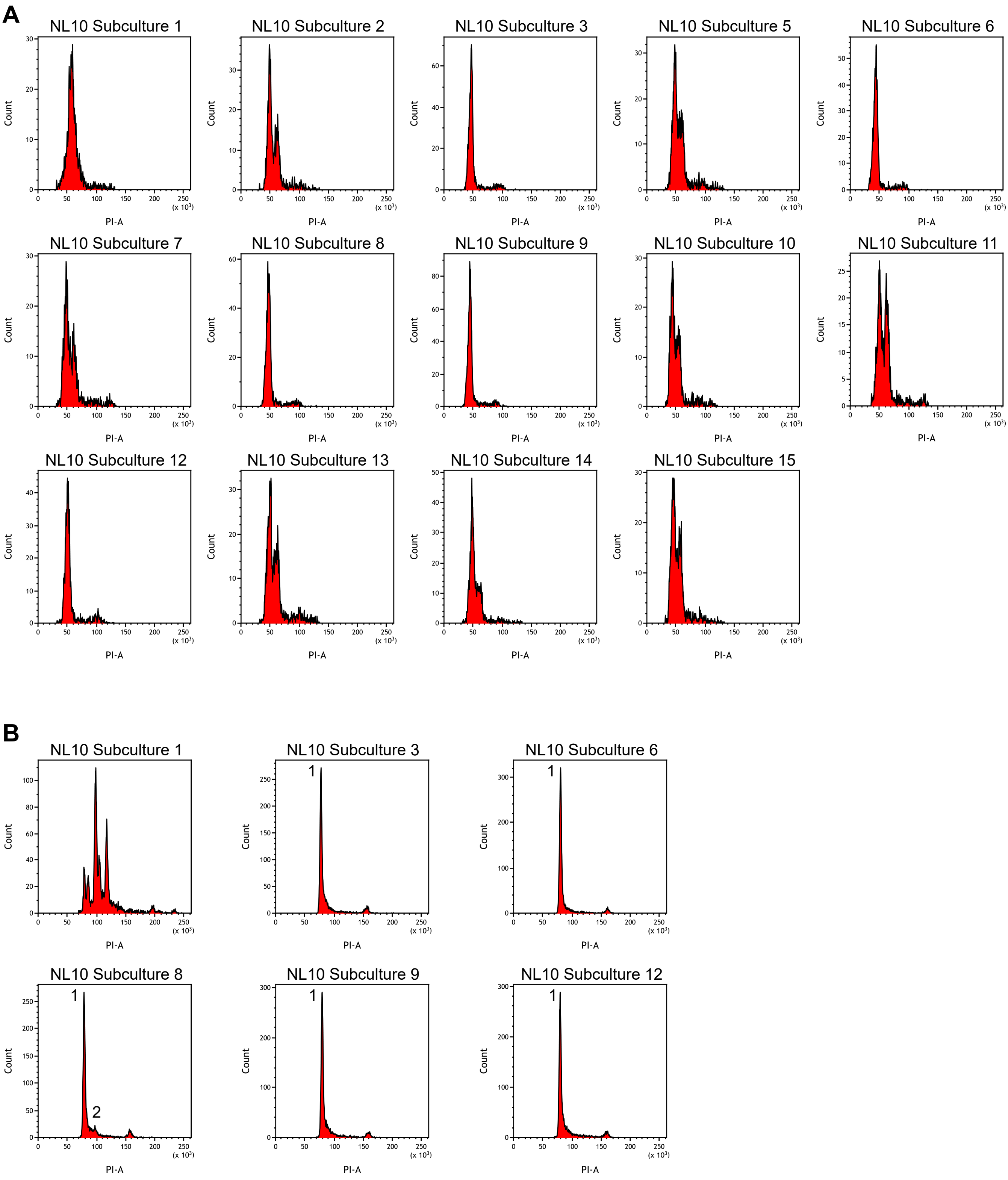

Fig. S1. Selection of NL10 subcultures to create NL12S. (A) Propidium Iodide Area (PI-A)-based histograms of 14 NL10 subcultures. Several subcultures have additional peaks representing karyotype polymorphisms. As these measurements are performed on a small number of worms (<100), different peaks are less distinct from each other than in other experiments. (B) The subcultures that showed a single peak were analyzed again 6 weeks later. Only four subcultures (3; 6; 9; and 12) still showed a single peak and were merged into a new NL12S culture with a single $2n = 8$ karyotype.

Biology Open • Supplementary information

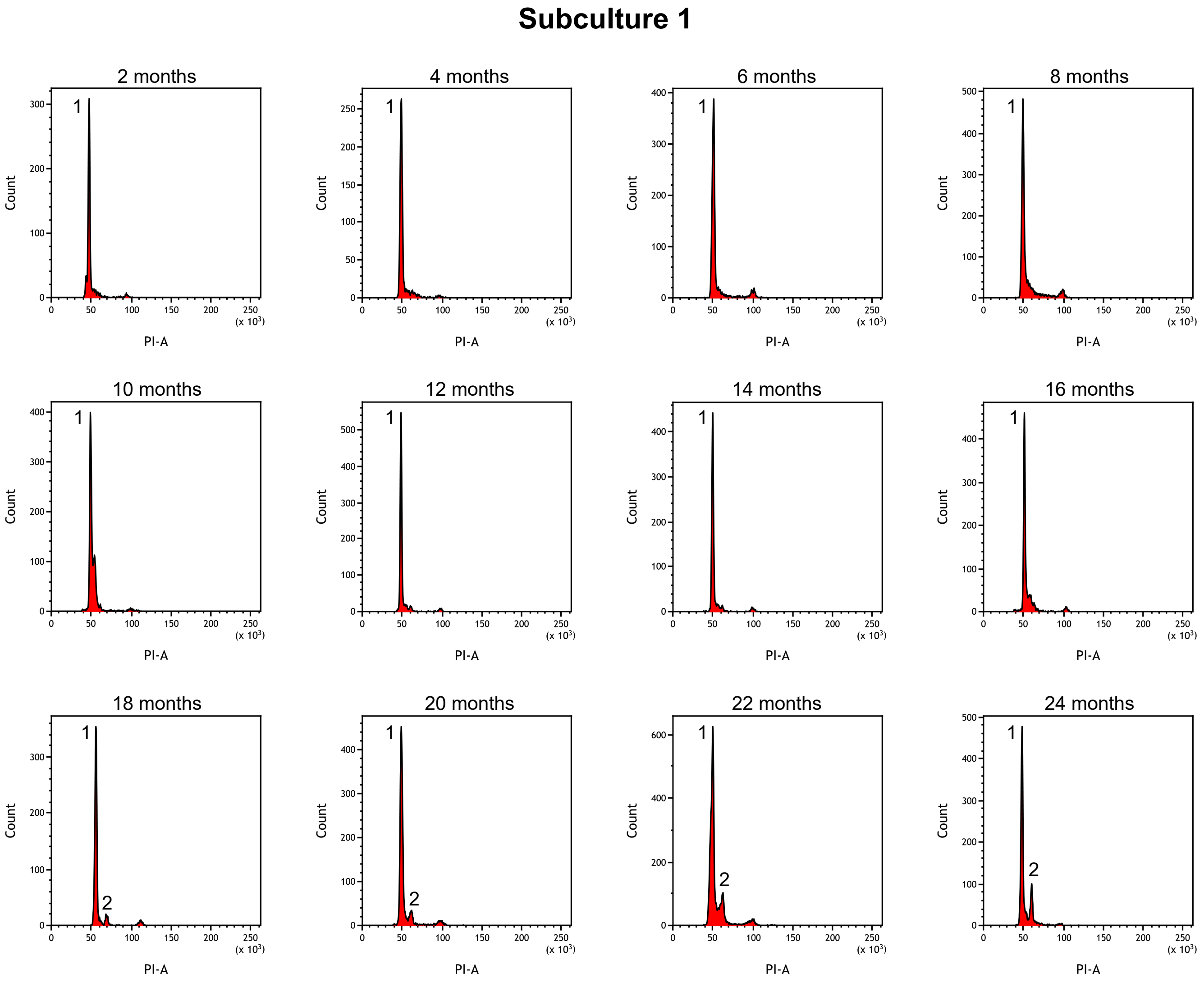

Fig. S2. Karyotype dynamics of NL12S subculture 1 over a period of two years. The subculture was analysed every 2 months and data are visualised as (PI-A)-based histograms. The number of peaks is indicated: 1 represents the *2n = 8* karyotype, and 2 represents the *2n = 9* karyotype.

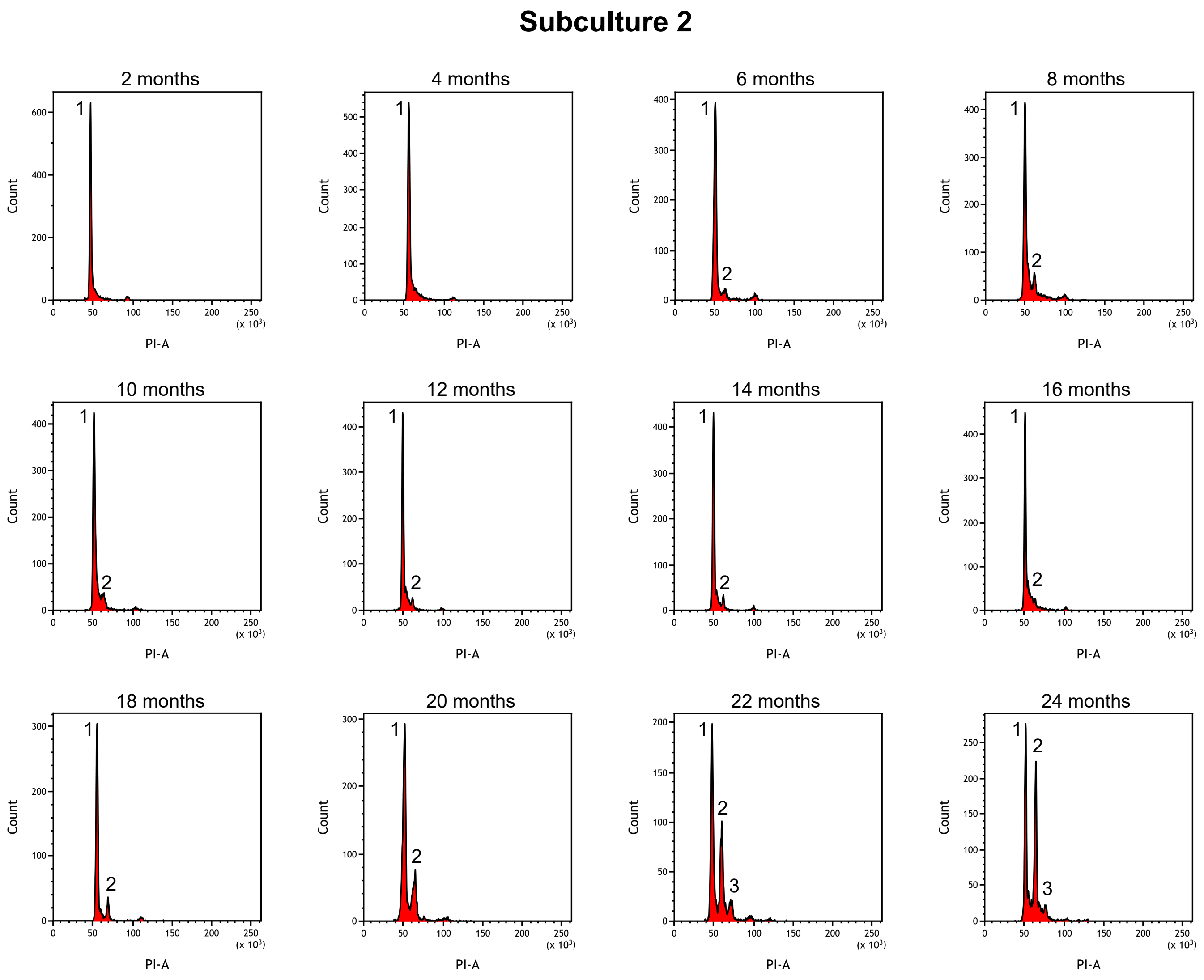

Fig. S3. Karyotype dynamics of NL12S subculture 2 over a period of two years. The subculture was analysed every 2 months and data are visualised as (PI-A)-based histograms. The number of peaks is indicated: 1 represents the *2n = 8* karyotype, 2 represents the *2n = 9* karyotype, and 3 represents the *2n = 10* karyotype. Note that the data for months 2, 6, and 22 are also represented in main Fig. 1.

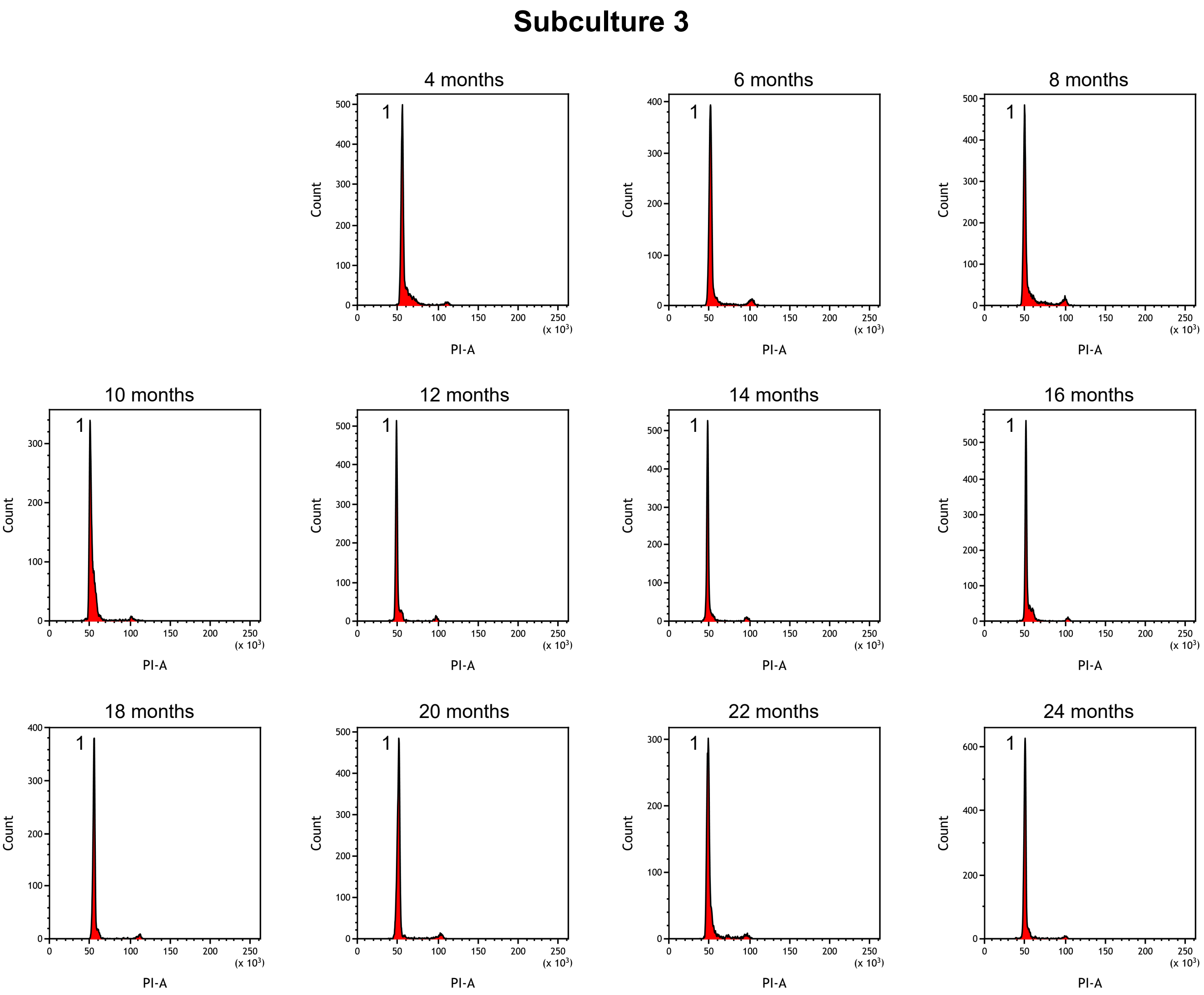

Fig. S4. Karyotype dynamics of NL12S subculture 3 over a period of two years. The subculture was analysed every 2 months and data are visualised as (PI-A)-based histograms. This culture did not obtain chromosome duplications, and is characterized by a *2n = 8* karyotype for the complete period.

Subculture 4

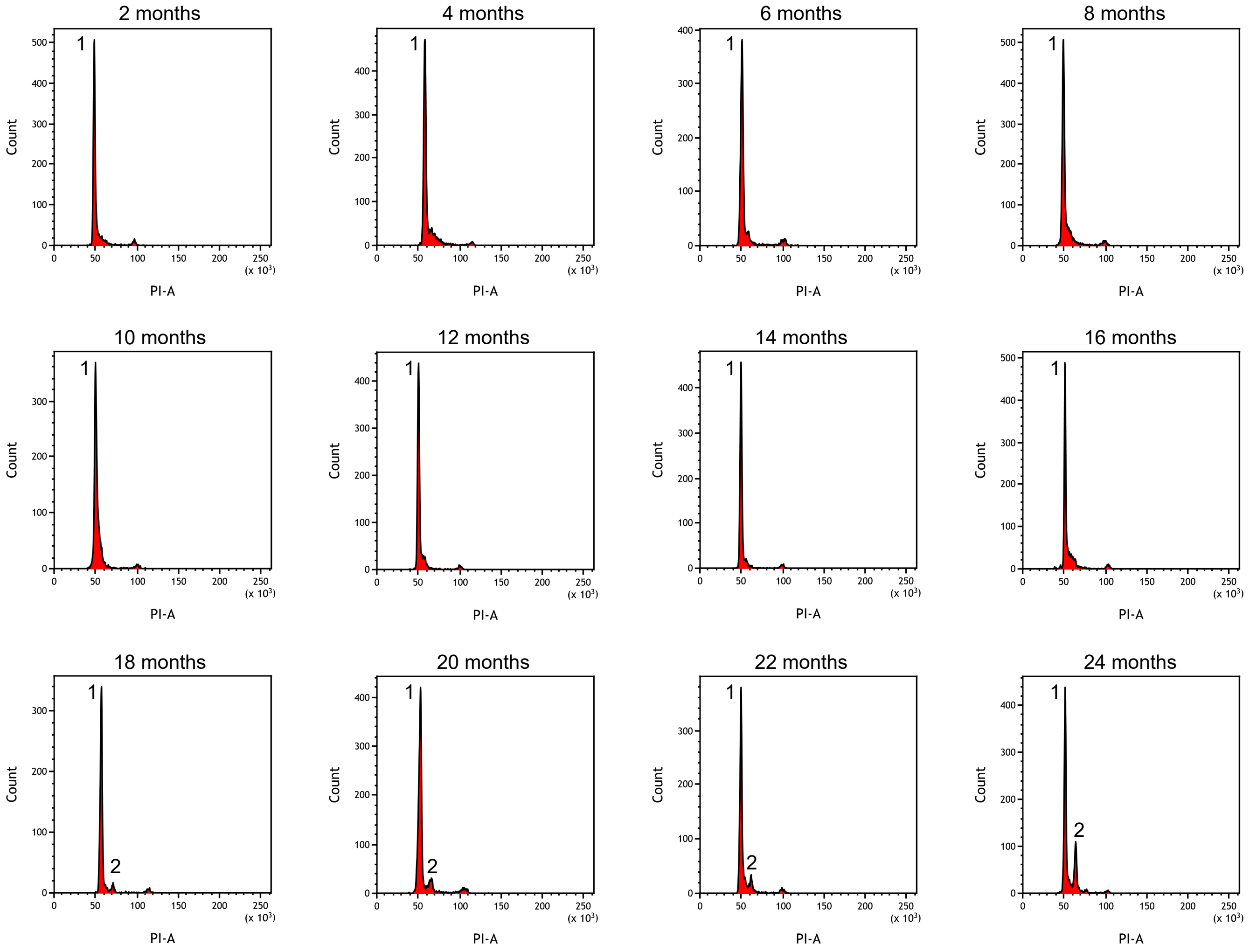

Fig. S5. Karyotype dynamics of NL12S subculture 4 over a period of two years. The subculture was analysed every 2 months and data are visualised as (PI-A)-based histograms. The number of peaks is indicated: 1 represent the *2n = 8* karyotype, and 2 represent the *2n = 9* karyotype.

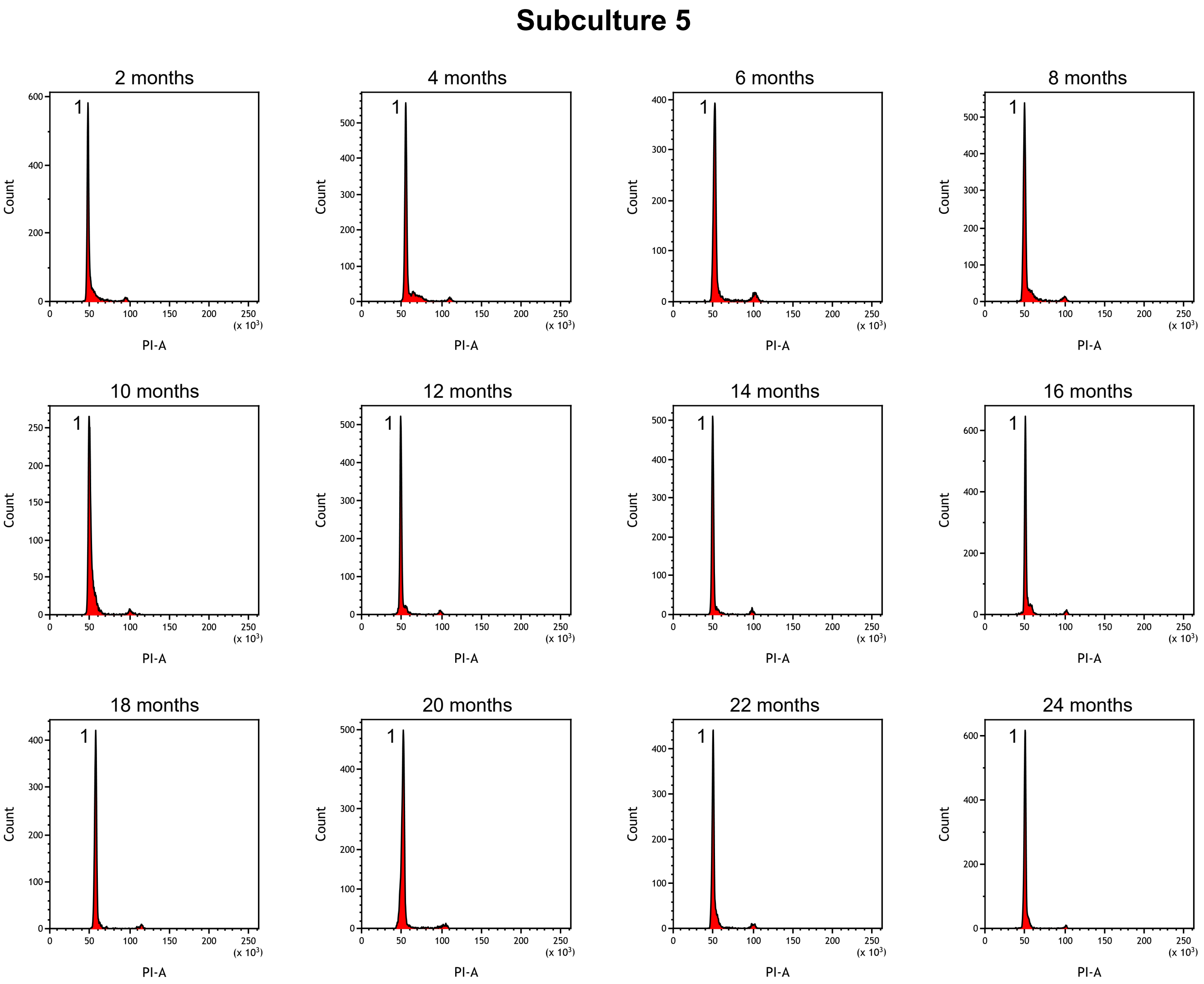

Fig. S6. Karyotype dynamics of NL12S subculture 5 over a period of two years. The subculture was analysed every 2 months and data are visualised as (PI-A)-based histograms. This culture did not obtain chromosome duplications, and is characterized by a *2n = 8* karyotype for the complete period.

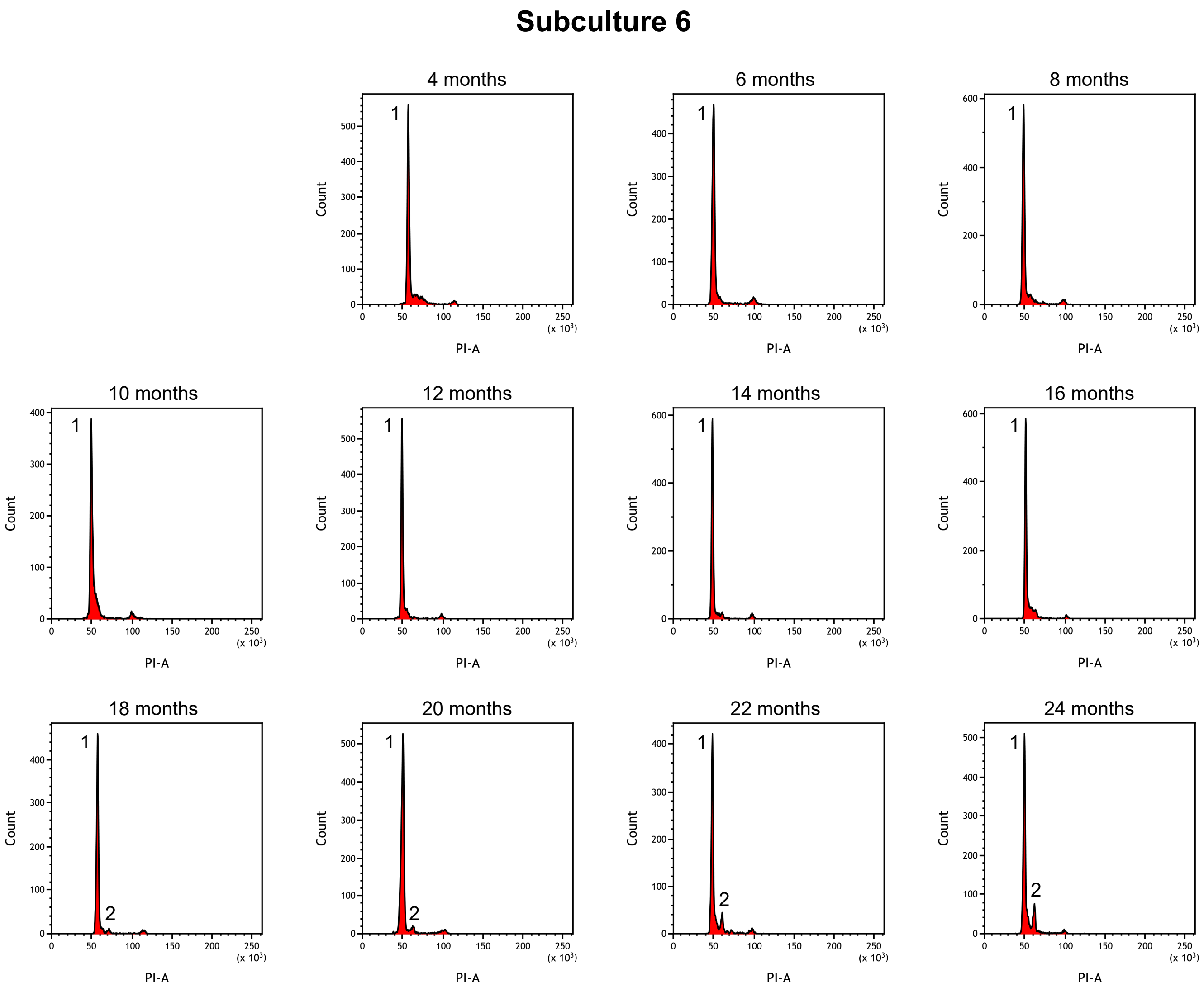

Fig. S7. Karyotype dynamics of NL12S subculture 6 over a period of two years. The subculture was analysed every 2 months and data are visualised as (PI-A)-based histograms. The number of peaks is indicated: 1 represent the $2n = 8$ karyotype, and 2 represent the $2n = 9$ karyotype.

Biology Open • Supplementary information

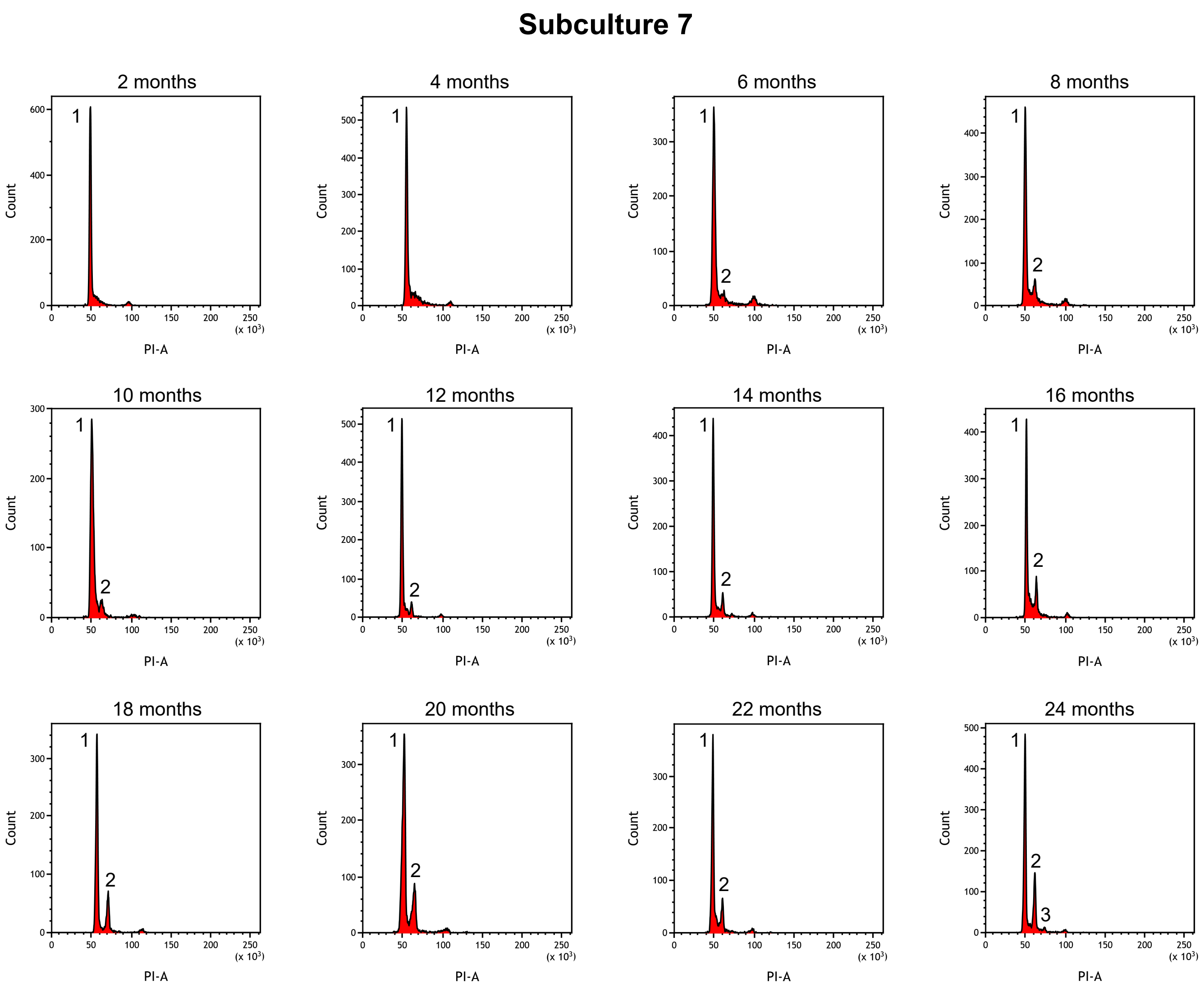

Fig. S8. Karyotype dynamics of NL12S subculture 7 over a period of two years. The subculture was analysed every 2 months and data are visualised as (PI-A)-based histograms. The number of peaks is indicated: 1 represent the *2n = 8* karyotype, 2 represent the *2n = 9* karyotype, 3 represent the *2n = 10* karyotype. Note that the data for the sixth month is also represented in main Fig. 1.

Biology Open • Supplementary information

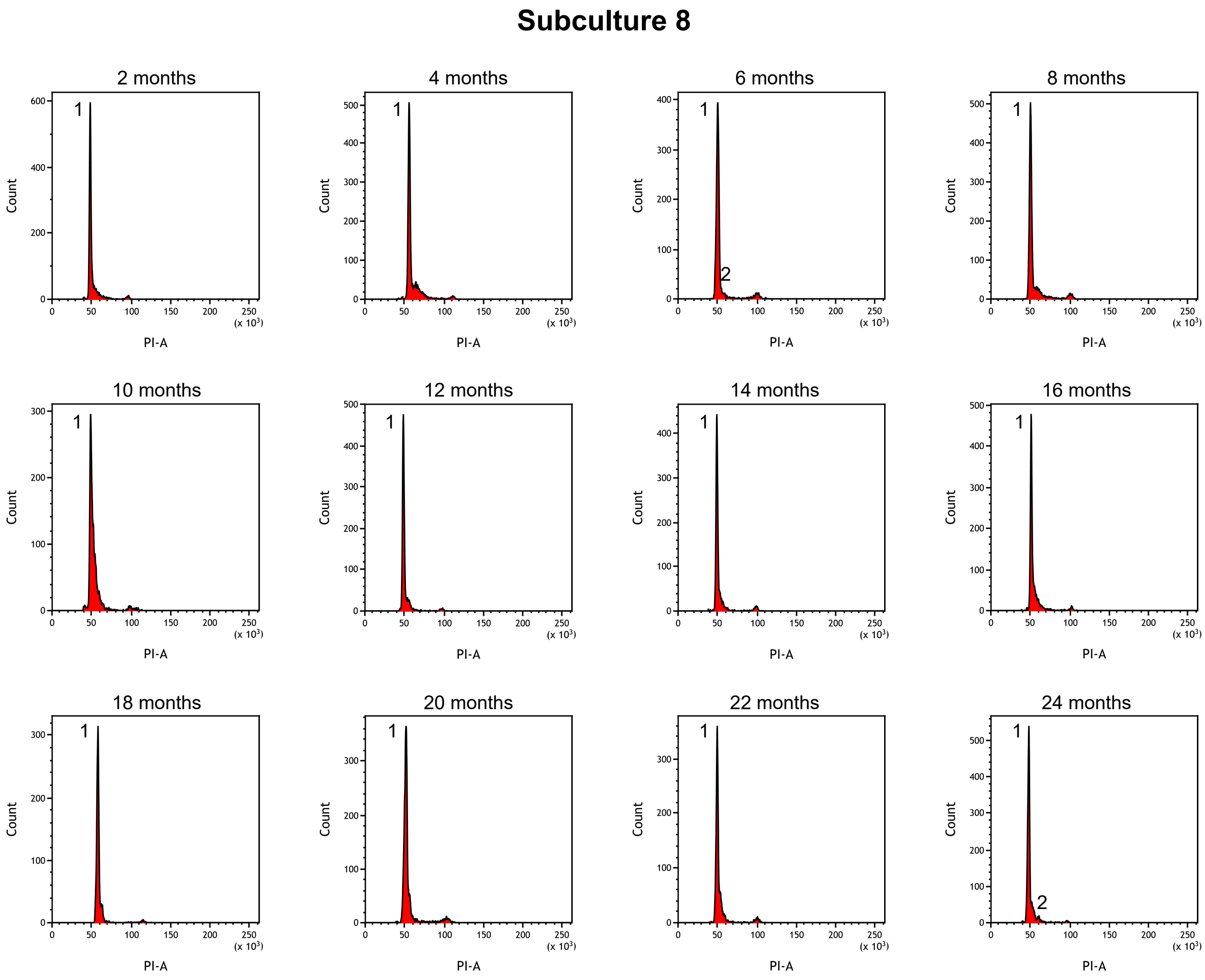

Fig. S9. Karyotype dynamics of NL12S subculture 8 over a period of two years. The subculture was analysed every 2 months and data are visualised as (PI-A)-based histograms. The number of peaks is indicated: 1 represent the *2n = 8* karyotype, and 2 represent the *2n = 9* karyotype.

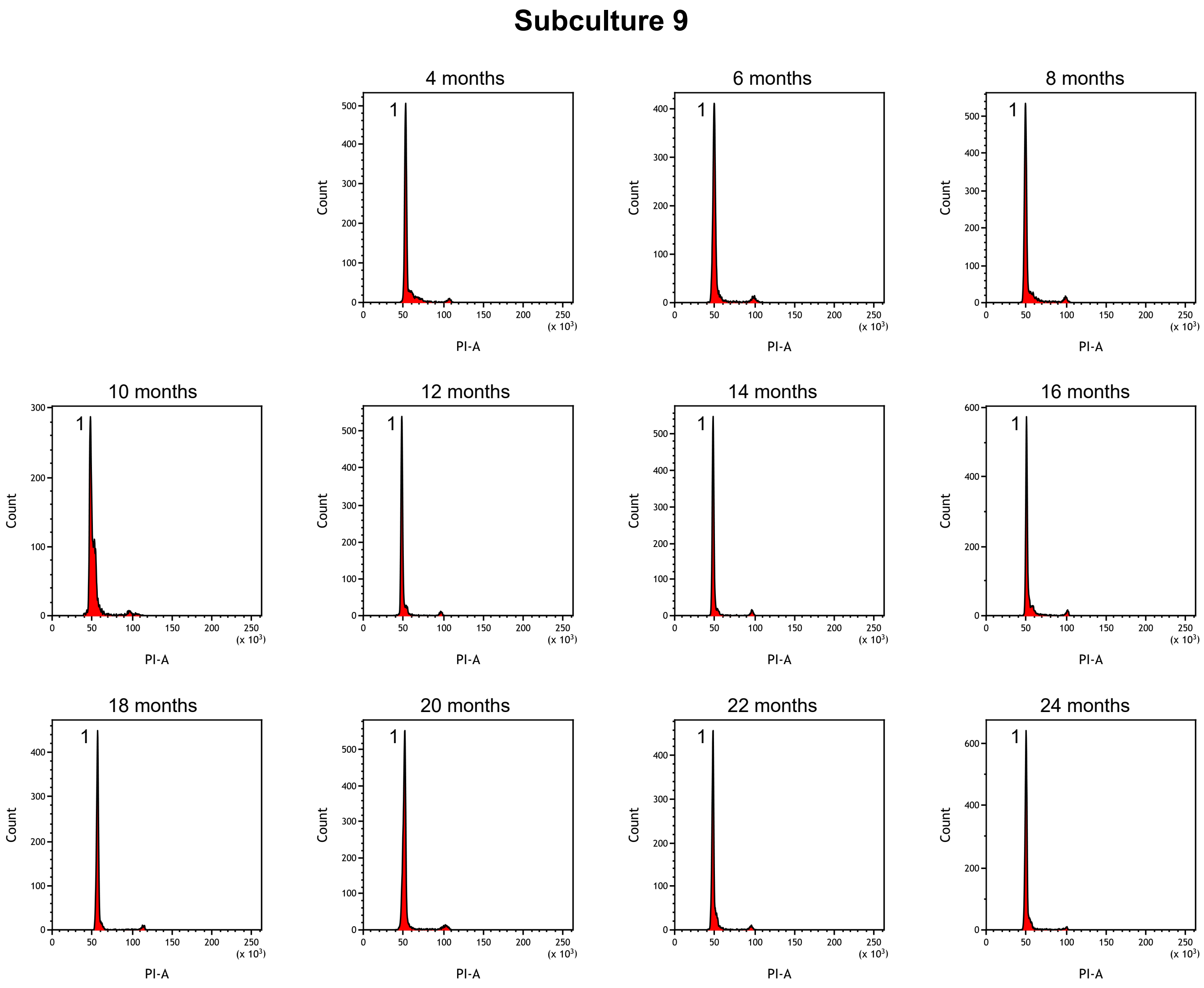

Fig. S10. Karyotype dynamics of NL12S subculture 9 over a period of two years. The subculture was analysed every 2 months and data are visualised as (PI-A)-based histograms. This culture did not obtain chromosome duplications, and is characterized by a $2n = 8$ karyotype for the complete period.

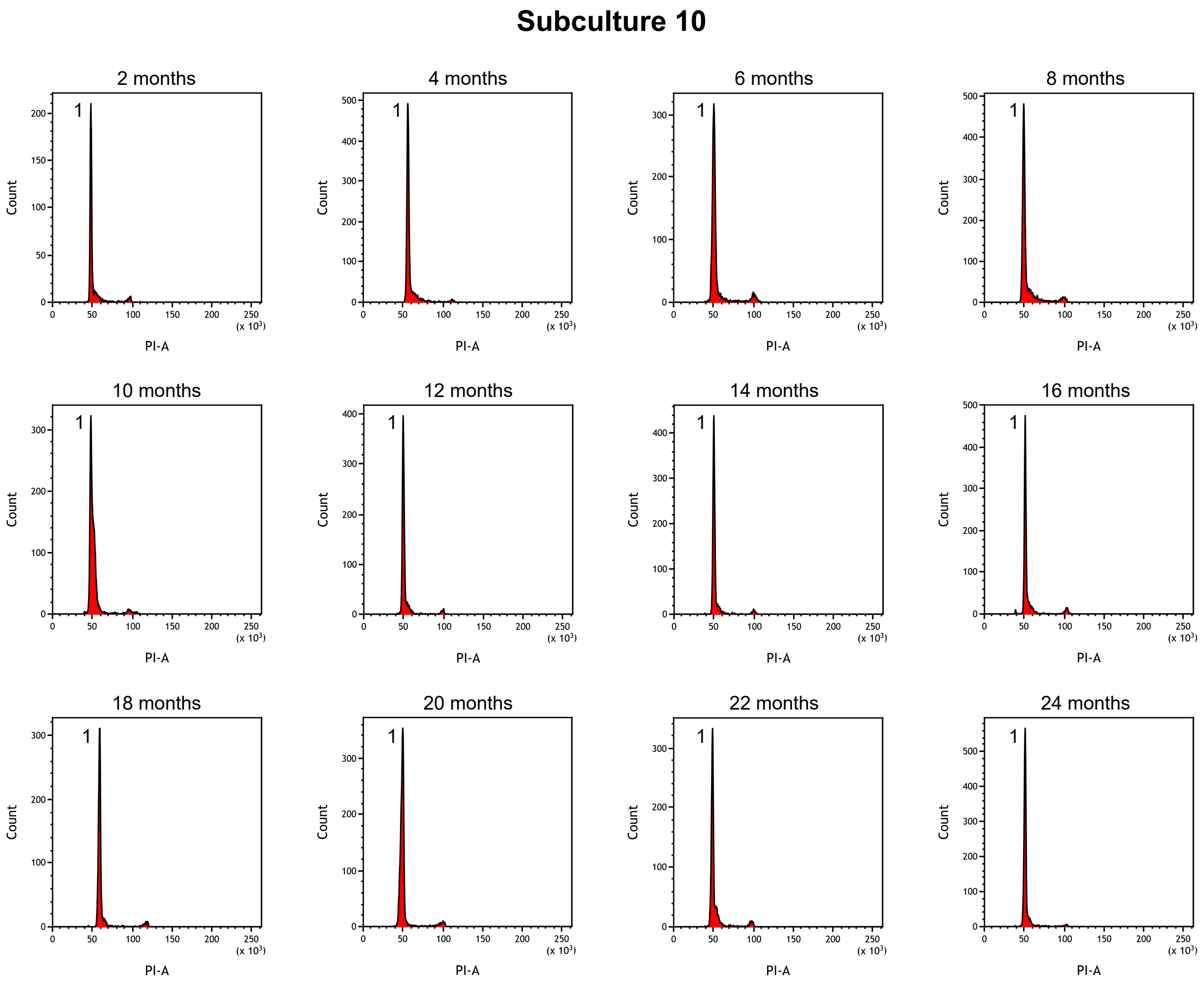

Fig. S11. Karyotype dynamics of NL12S subculture 10 over a period of two years. The subculture was analysed every 2 months and data are visualised as (PI-A)-based histograms. This culture did not obtain chromosome duplications, and is characterized by a *2n = 8* karyotype for the complete period.

Biology Open • Supplementary information

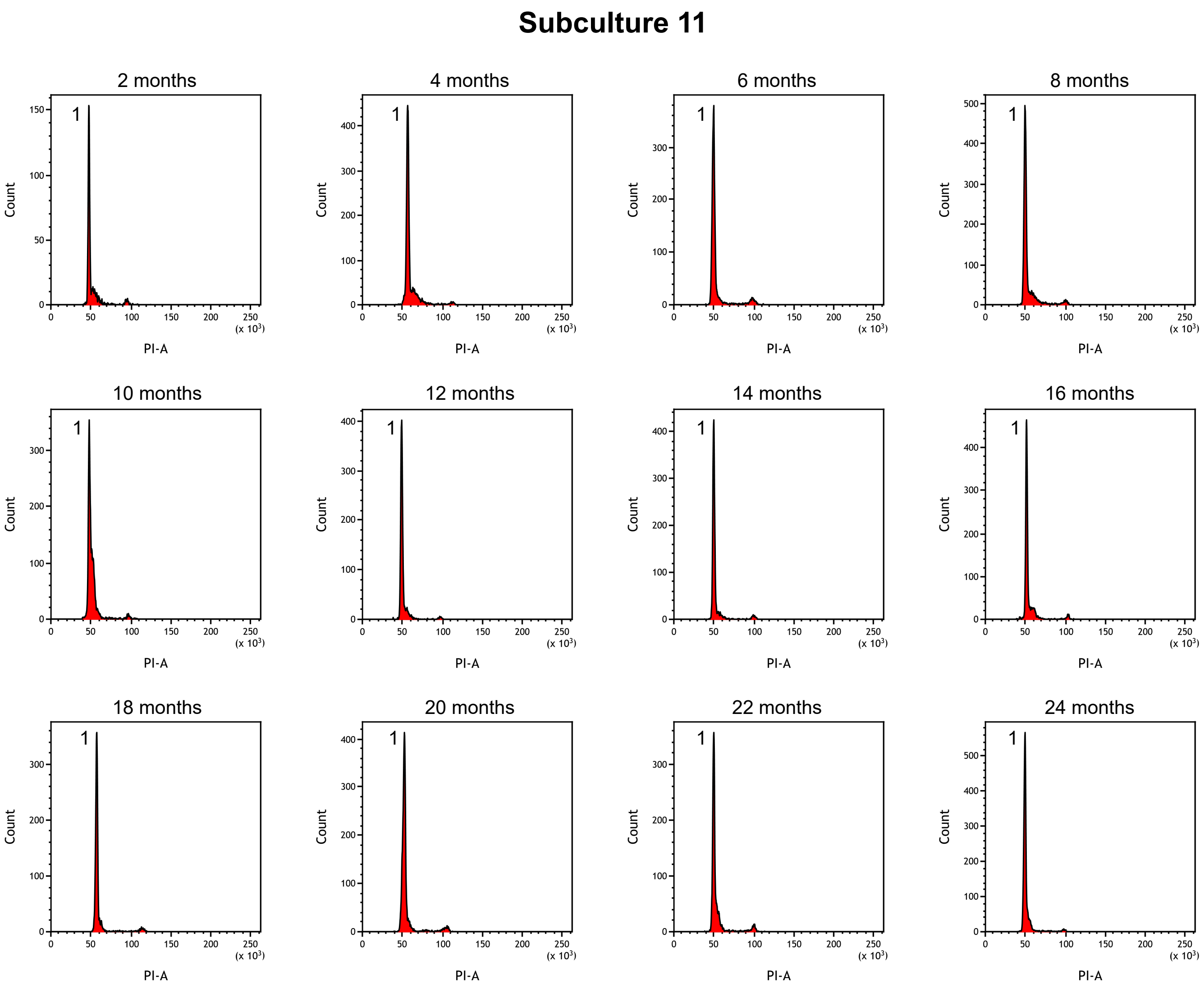

Fig. S12. Karyotype dynamics of NL12S subculture 11 over a period of two years. The subculture was analysed every 2 months and data are visualised as (PI-A)-based histograms. This culture did not obtain chromosome duplications, and is characterized by a *2n = 8* karyotype for the complete period.

Biology Open • Supplementary information

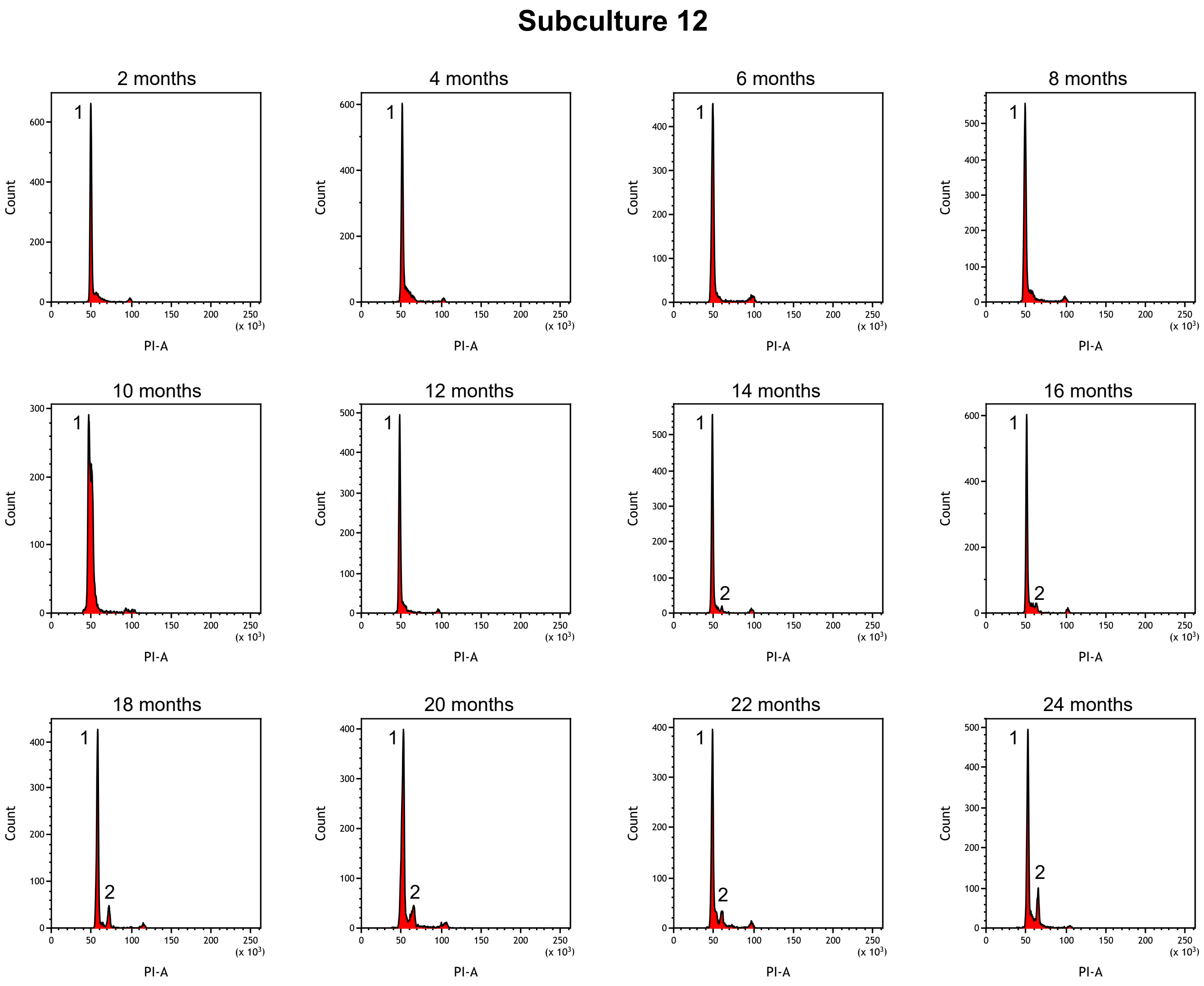

Fig. S13. Karyotype dynamics of NL12S subculture 12 over a period of two years. The subculture was analysed every 2 months and data are visualised as (PI-A)-based histograms. The number of peaks is indicated: 1 represent the *2n* = *8* karyotype, and 2 represent the *2n* = *9* karyotype.

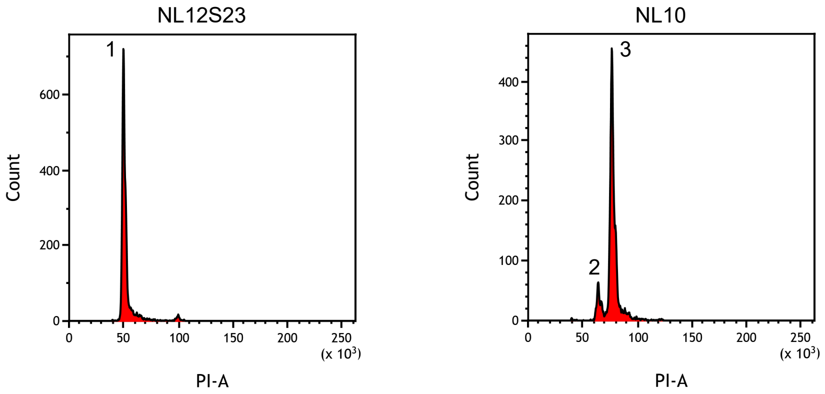

Fig. S14. Long-term karyotype dynamics. The NL12S23 culture, as a reference for a culture with only the *2n = 8* karyotype, represented as a single peak (1), located at the 50 value on the X-axis. The NL10 culture after 11 years of culturing without interfering with the karyotype. The *2n = 8* and the corresponding peak (1) completely disappeared. There is a small peak (2) representing the *2n = 9* karyotype, and a large peak (3) representing the *2n = 10* karyotype. This suggests that, in the long run, worms with the *2n = 10* karyotype outcompete the others.

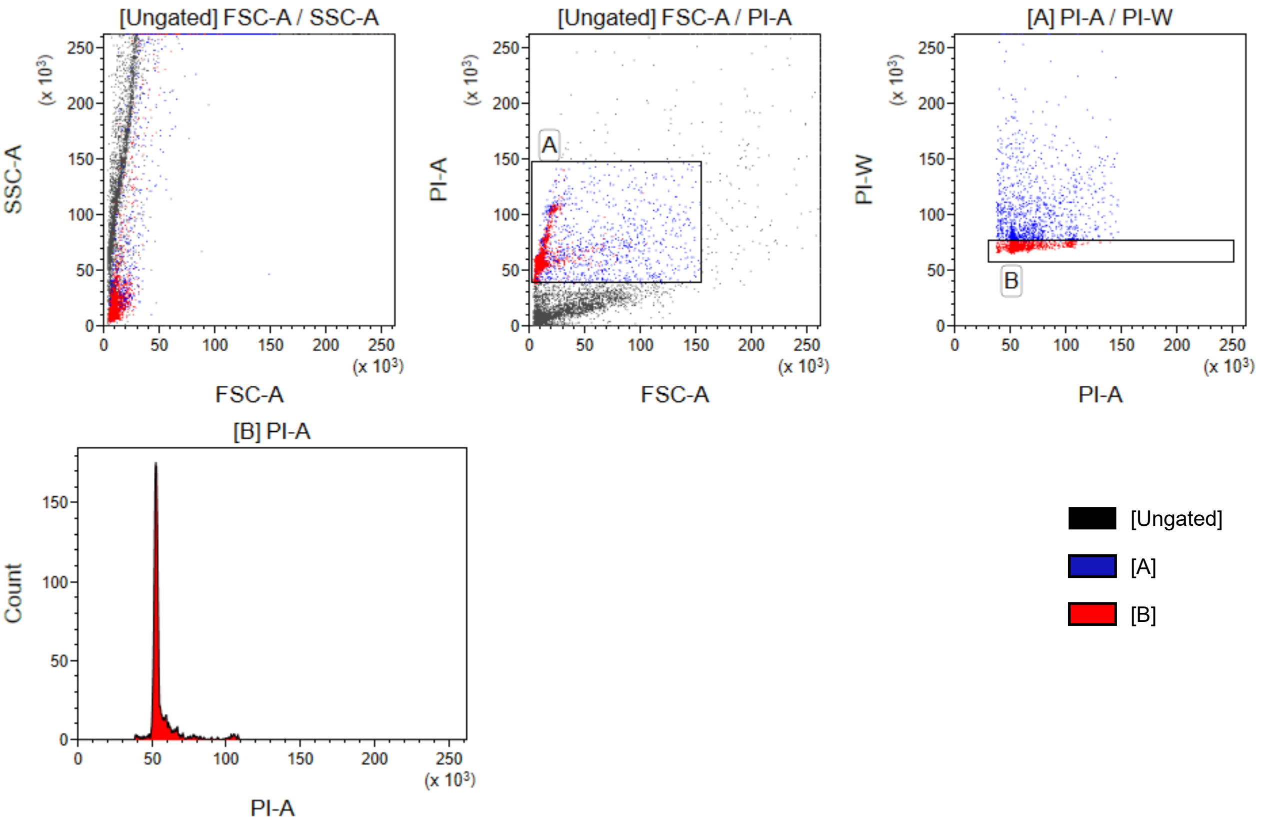

Fig. S15. Kaluza data sheet showing the gating strategy.

This figure shows a screenshot of a Kaluza worksheet. In all plots, events present in gate A are shown in blue, events present in gate B are shown in red, and the ungated events are shown in black. The first plot visualizes the Side Scatter Area (SSC-A), representing the internal complexity on the Y-axis, and the Forward Scatter Area (FSC-A), representing the size on the X-axis. This plot is not used for the gating strategy, but visualizes the nuclei (in red and blue) versus debris (in black). The second plot visualizes the PI-Area (A) on the Y-axis and the FSC-A on the X-axis. Gate A is drawn to select PI-positive nuclei. The events below the gate represent unlabelled debris. The gated nuclei are visualized in the third plot. The PI-Width (W) on the Y-axis represents the time a particle takes to pass through the laser. The X-axis visualizes PI-A, which represents the total fluorescence of the particle. Gate B is drawn by focusing on events with low PI-W values, as they represent single nuclei. The single nuclei are visualized in the PI-A histogram. The Count on the Y-axis represents the number of events measured. We often adjust the laser power to position the large peak round the 50-value on the X-axis to facilitate the comparison of samples measured at different times. The second small peak of approximately 100 represents cells in the G2 and Mitotic phases of the cell cycle.

Biology Open • Supplementary information
